# Dopamine synergizes with caffeine to increase the heart rate of *Daphnia*

**DOI:** 10.12688/f1000research.12180.1

**Published:** 2018-03-01

**Authors:** Aman Kundu, Gyanesh Singh

**Affiliations:** 1 Fs Convent School, Jind, Hariyana, 126113, India; 2 School of Bioengineering and Biosciences, Lovely Professional University, Phagwara, Punjab, 144411, India

**Keywords:** Dopamine, heart, neurotransmitter, cardiac, central nervous system

## Abstract

Dopamine is a key neurotransmitter, and is widely used as a central nervous system (CNS) agent.  Dopamine plays an important role in humans, including a major role in reward and motivation behaviour. Several addictive drugs are well known to increase neuronal dopamine activity. We selected *Daphnia*, an important model organism, to investigate the effect(s) of selected CNS agents on heart rate. Dopamine’s effects on *Daphnia*’s heart has not been previously reported. Caffeine is a well-known and widely consumed stimulant. Ethanol is well known for its effects on both neurological and physiological processes in mammals. We tested the effect of dopamine on the heart rate of *Daphnia*, and compared its effect with caffeine and ethanol alone and in combination. Both caffeine and dopamine were found to instantly increase the heart rate of *Daphnia* in a dose-dependent manner. Interestingly, caffeine synergized with dopamine to increase *Daphnia*’s heart rate. As ethanol decreased the heart rate of *Daphnia *and dopamine increased the heart rate of *Daphnia, *we wanted to test the effect of these molecules in combination *.* Indeed, Dopamine was able to restore the ethanol-induced decrease in the heart rate of *Daphnia*.  Effects of these CNS agents on *Daphnia *can possibly be correlated with similar effects in the case of mammals.

## Introduction

Neurotransmitters are the key mediators of communication between nerve cells. Because of their effect on brain and spinal cord, central nervous system (CNS) agents can be used to control or treat variety of medical conditions ^1^. Stimulation of the hypothalamus can lead to cardiovascular disturbances, indicating a direct connection between the heart and the CNS ^2, 3^. Different types of rewards are known to increase the level of dopamine in the brain ^4^. *Daphnia* are small crustaceans commonly known as “water fleas”, and are found in water bodies ^5^. *Daphnia* is an ideal organism for research, as it has short life span, and can easily be cultured ^6^. These organisms can feed on algae, yeast and bacteria ^5^. More importantly, *Daphnia* are transparent, thus allowing clear visualization of different organs, including the heart ^7^. The organs are protected by a thin membrane that allows the penetration of different compounds; therefore assisting with heart rate monitoring in real time ^5^. Using a microscope that has computer-aided real-time imaging capabilities, the effect of various compounds can be observed on *Daphnia‘s* heart in real time. *Daphnia’s* life span is 40–50 days, which varies in different species and also changed with environmental conditions, especially temperature. Male and female *Daphnia* can easily be differentiated, as female *Daphnia* have brood pouch that holds eggs. These eggs develop into embryos, leading to the production of juveniles that attain sexual maturity within ten days.

Dopamine is important for normal cardiopulmonary response to exercise and is necessary for optimal high-intensity exercise performance. Blocking dopamine receptors appears to be detrimental to exercise performance ^8^. Caffeine, by antagonizing adenosine A2A receptors, is known to augment dopamine signalling in the brain ^9, 10^. Even at routine doses, caffeine can enhance dopamine receptor accessibility in the mammalian CNS ^10^. Caffeine has also been reported to normalize the heart rate of *Daphnia*, which is decreased by atropine and atenolol ^11^. Ethanol is known to cause progressive weakness, difficulty in walking, and lowered heart rate ^12^. Ethanol also inhibits calcium dependent neurotransmitter release, and, excitatory and inhibitory postsynaptic potentials in cultured spinal cord neurons ^13^.

The aim of the present study was to investigate the effect of Dopamine on *Daphnia’s* heart rate, alone and in combination of caffeine and ethanol. The rationale behind this research was that both caffeine and ethanol are known to affect nervous system functions ^14^, and dopamine is a major neurotransmitter.

## Methods

### *Daphnia* culture

*Daphnia* were isolated from Chitti Vai river of Punjab. For the isolation of *Daphnia*, 0.5–2.0 litres of river water was collected and transported to laboratory. Adult *Daphnia* were manually identified as per the standard identification features ^15^, and filtered out using muslin cloth. These adults were cultured in 300 ml glass jars containing river water that was filtered with muslin cloth. *Daphnia* culture was supplemented with 0.5% yeast culture, added every third day. Yeast culture, in this case, was used as a food for *Daphnia*. Algae, yeast or bacteria are preferred food for *Daphnia*. Although, many workers use river water for *Daphnia* culture presuming that it would have better mineral composition, in our case, we were also able to culture *Daphnia* in aged tap water in the similar manner. Cultures were routinely monitored to ensure production of healthy *Daphnia*.

### Counting of *Daphnia‘s* heart rate

To investigate the effect of certain agents on the heart rate of *Daphnia*, real-time monitoring of changes in the heart rate of *Daphnia* is required. We used a microscope equipped with computer-aided real-time imaging capability (Magnus Live usb camera viewer, version 2.0, Magnus Analytics, New Delhi-110044, India), and for each reading heart rate was initially counted without any treatment (control). Subsequently, changes in the heart rate was monitored after the addition of selected agents. Each *Daphnia* was placed on the glass slide with 100 ul of water. The slide was observed in real time under the microscope at 40x or 100x magnification, and heart rate was counted. To avoid the effect of temperature or other environmental factors, counting was done after five seconds of starting the microscope. Subsequently, the microscope was switched off for five seconds, cardiovascular agents were added (see Table 1), and heart rate was counted again.

**Table 1.** Central nervous system agents used.

Name (source)	Concentrations
Caffeine (Loba Chemie Pvt Ltd, Mumbai, India)	0.08 to 0.32 mg/ml
Dopamine (Amrit Pharmaceuticals, Aurangabad, India)	0.4 to 3.2 mg/ml
Ethanol (Himedia Laboratories, Mumbai, India)	2–8%

### Statistical analysis

A paired t test analysis was done to compare changes in heart rates upon treatment with different agents. Statistical analyses were performed using GraphPad Prism version 6.00 for Windows (GraphPad Software, San Diego, CA, USA). P<0.05 was considered significant.

## Results and discussion

### Dopamine, like caffeine, increases the heart rate of *Daphnia* in a dose-dependent manner

**Figure 1.**
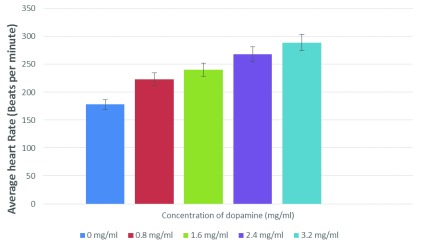
Dopamine increases the heart rate of *Daphnia* in a dose-dependent manner. This experiment was performed two times, and a paired t test analysis vs control indicated the following P values: 0.0070 (for 0.8 mg/ml), 0.0255 (1.6 mg/ml), 0.0424 (2.4 mg/ml), and 0.0344 (3.2 mg/ml). These values are statistically significant.

**Figure 2.**
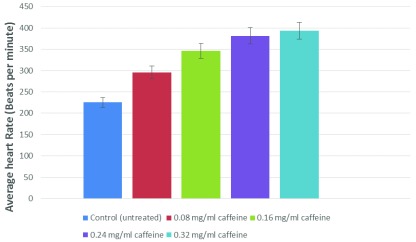
Caffeine increases the heart rate of *Daphnia* in a dose-dependent manner. This experiment was done two times, and a paired t test analysis vs control revealed the following P values: 0.0406 (0.08 mg/ml), 0.0263 (0.16 mg/ml), 0.0367 (0.24 mg/ml), and 0.0189 (0.32 mg/ml). These values are statistically significant.

Dopamine’s effects on *Daphnia*’s heart has not been reported previously. We hereby report that dopamine instantly increases the heart rate of *Daphnia* in a dose-dependent manner, and a significant increase (25.7%) in the heart rate was observed, even at a low dose of 0.8 mg/ml ( Figure 1). Caffeine showed a similar effect on *Daphnia*’s heart rate at a 10-times lower concentration than dopamine (28.5% increase at 0.08 mg/ml, Figure 2). Dopamine is the precursor of norepinephrine, and has been shown to augment heart activity by affecting beta-adrenergic receptors, in the case of a canine model ^16^. Furthermore, dopamine can cause both relaxation and contraction of vascular smooth muscle. Dopamine is also known to augment heart activity, pulmonary pressure, and cardiac index in the case of normal and hypertensive individuals ^17^.

### Dopamine synergizes with caffeine to increase the heart rate of *Daphnia*

**Figure 3.**
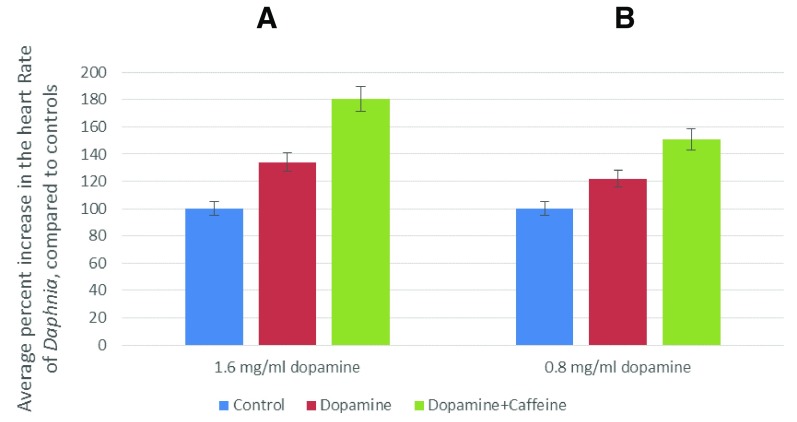
Dopamine synergizes with caffeine to increase the heart rate of *Daphnia*. *Daphnia*’s heart rate was measured upon treatment with dopamine alone (red) or a combination of dopamine and caffeine (green). The concentration of caffeine (in combination with dopamine) was ( **A**) 40 ug/ml and ( **B**) 120 ug/ml. This experiment was performed two times, and a paired t test analysis vs control indicated the following P values: 0.0374 (0.8 mg/ml dopamine) and 0.0230 (1.6 mg/ml dopamine). These values are statistically significant.

Caffeine, in combination with dopamine, increased *Daphnia*’s heart rate more than when the agents were administered alone, which suggests a synergistic activity ( Figure 3). Dopamine has also been previously reported to play a role in the responses of *Drosophila* to cocaine, nicotine or ethanol ^18^.

### Dopamine overcomes an ethanol-induced decrease of the heart rate of *Daphnia*

**Figure 4.**
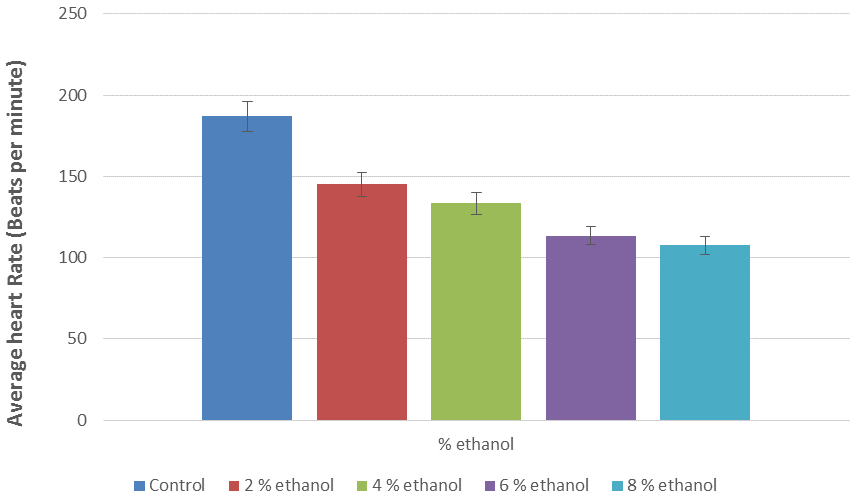
Effect of ethanol on the heart rate of *Daphnia*. This experiment was done two times, and a paired t test analysis vs control indicate the following P values: 0.0152 (2% ethanol), 0.0059 (4% ethanol), 0.0130 (6% ethanol), and 0.0280 (8% ethanol). These values are statistically significant.

To see the effect on the heart rate of *Daphnia*, ethanol was used at a concentration ranging from 2–8%, and was found to decrease the heart rate of *Daphnia* in a dose-dependent manner ( Figure 4).

**Figure 5.**
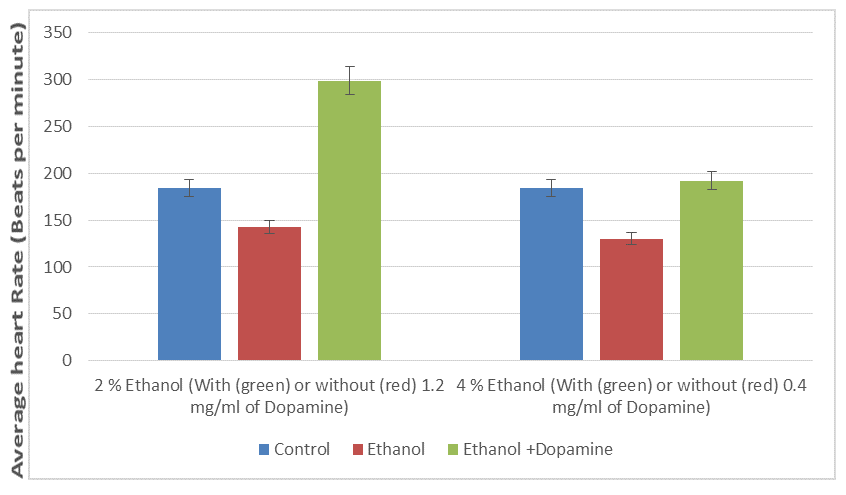
Dopamine overcomes the ethanol-induced decrease on the heart rate of *Daphnia*. At 2% ethanol, dopamine-induced increase in the heart rate was 62.5% compared to control, and 84.8% compared to ethanol-induced heart rate. At 4% ethanol, dopamine-induced increase in the heart rate was 4.3% compared to control, and 33.7% compared to ethanol-induced heart rate.

We observed that dopamine was able to rescue the ethanol-induced decrease in the heart rate of *Daphnia*, even at a concentration of 0.4 mg/ml ( Figure 5).

Effect of dopamine, caffeine and ethanol on the heart rate of *Daphnia* Click here for additional data file. Heart rates (beats per minute) was initially counted without any treatment (controls). Subsequently, changes in the heart rate was monitored after the addition of selected agents. Copyright: © 2018 Kundu A and Singh G 2018 Data associated with the article are available under the terms of the Creative Commons Zero "No rights reserved" data waiver (CC0 1.0 Public domain dedication).

## Conclusion

This fundamental investigation can be of enormous importance, as caffeine and ethanol are the most widely consumed psychoactive drugs, and dopamine is a master neurotransmitter that is known to be involved in variety of diseases ^19, 20^. It is possible that these psychoactive agents can have similar or more drastic effects in humans. It is, therefore, very important to urgently investigate the effect of these psychoactive agents, alone or in combination, in humans. Such studies can provide crucial information that can be used in a variety of clinical settings. For example, cases of alcohol or caffeine intoxication can be managed by dopamine therapy, treatment(s) of cardiac disorders may be different for alcoholics or coffeeholics, and patients undergoing dopamine therapy need to be regularly monitored for cardiothoracic status, and alcohol/caffeine consumption.

## Data availability

The data referenced by this article are under copyright with the following copyright statement: Copyright: © 2018 Kundu A and Singh G

Data associated with the article are available under the terms of the Creative Commons Zero "No rights reserved" data waiver (CC0 1.0 Public domain dedication). 

Dataset 1: Effect of dopamine, caffeine and ethanol on the heart rate of *Daphnia*. Heart rates (beats per minute) was initially counted without any treatment (controls). Subsequently, changes in the heart rate was monitored after the addition of selected agents. DOI, 10.5256/f1000research.12180.d194189 ^21^
