# Supplementary material for: Dopamine synergizes with caffeine to increase the heart rate of Daphnia
Source: F1000Res. 2018 Mar 1;7:254. [Version 1] doi: 10.12688/f1000research.12180.1 (PMC6024234; doi:10.12688/f1000research.12180.1)
Supplement: Peer review history

# Peer review: Referee response for version 1

**DOI:** 10.5256/f1000research.13184.r35079

**Published:** 2018-06-27

**Authors:** Cecilia Scorza

**Affiliations:** 1 Department of Experimental Neuropharmacology, Clemente Estable Institute of Biological Research (IIBCE), Montevideo, Uruguay

The study “Dopamine synergizes with caffeine to increase the heart rate of Daphnia” done by Aman Kundu and Gyanesh Singh, investigates the effect of dopamine, caffeine and alcohol on Daphnia’s heart rate. Also the combination of caffeine and ethanol was tested. The rationale of the paper was to use the Daphnia’s heart rate to test treatment(s) of cardiac disorders in high consumers of coffee or alcohol. The importance and objectives of the study are well explained and methods are well described, although some aspects of this manuscript should be really improved and corrected. 

 The statistical Analysis applied is not correct; consequently I have some doubts if the results would be the same applying a correct statistical analysis. Authors used t-paired test to evaluate a dose-dependence curve in which more than two concentrations are compared. I suggest to authors apply One-Way ANOVA followed by a post hoc test (for example, Newman-Keuls). Additionally, authors indicated that the experiments were done two times. I understand that this means that a duplicate of each experiment were performed, is it correct?. If this is the case, a mean ± SD should be used to compare data. Please, correct.

 Another important issue is about the synergism. Author concluded that dopamine synergizes with caffeine to increase the heart rate of Daphnia. But, synergism should be declared if a sub-threshold concentrations of DA and Caff is used. If not, only a potentiation is achieved.

 Please, correct the terminology of synergism or other experiments should be done using the combination of sub-threshold doses of DA and Caff.

 It would be very important to add positive controls to test Daphnia’s heart rate, for example, noradrenaline or atropine, both are prototypical substances that increase heart rate in vertebrate animals.

 In the conclusion: in addition to the purpose of this kind of assay, I wondering if Daphnia’s heart rate assay could be more suitable as a biomarker of toxicity instead of serve as a screening test of different drugs to alter Daphnia’s heart rate?

I have read this submission. I believe that I have an appropriate level of expertise to confirm that it is of an acceptable scientific standard, however I have significant reservations, as outlined above.

# Peer review: Referee response for version 1

**DOI:** 10.5256/f1000research.13184.r31349

**Published:** 2018-04-13

**Authors:** Mrinal K. Poddar

**Affiliations:** 1 Department of Biochemistry, University of Calcutta, Kolkata, India

The original article written by Kundu and Singh focuses on the study of the effects of cardiovascular drugs of the heart rate of Daphnia. In this study the authors have used an interesting heart model of Daphnia.

 The article represents the synergistic effect of dopamine and caffeine to increase the heart rate in Daphnia, the tiny water fleas. Though, neither this model can be compare with mammals nor their organ function. The authors have tried to show some effects without mentioning the mechanism of effects. I think they should clarify the followings points:

 1. Authors have analyzed the data with a simple t test. Why they didn’t they approach with ANOVA test?  Moreover, statistical analysis with only two observations is not sufficient. Observations should be made of at least four times for significance test.

 2. In Figures, the vertical line given on top of each bar represents what? - SEM or SD?

 3. Table 1 shows the lowest concentration of dopamine as 0.4mg/ml which is missing in the entire manuscript. Where is the result of 0.4 mg/ml?

 4. Why did the authors choose 40µg or 120µg/ml instead of mg/ml that they have tested and represented in Figure 1. What is the reason of this sudden switch over? Nothing is clear to me!

 5. In Figure 3 Why the authors have used the combination of lower concentration of caffeine (40µg/ml) with higher concentration of dopamine (1.6mg/ml) and higher concentration of caffeine (120 µg/ml) with lower concentration of dopamine (0.8mg/ml)? This needs clarification.

 6. Figure 5 have the same queries like Figure 3 and it should be better in both the cases of combinations, keep one constant and vary the other one.

 7. The Daphnia model does not directly resemble the mammalian system. The conclusion they have made in the manuscript requires rethinking and should be presented accordingly.

 8. In the manuscript the authors didn’t mention about the treatment procedure. How have they administered the dopamine, caffeine or alcohol? What is the route of administration of these compounds? Are those administered or given in the culture medium? Depending on the route of administration for this particular model, the author should discuss about the mechanism of action of these agents on Daphnia heart rate or cardiovascular system. 

 Suggestions:

 I think for indexing following works are needed to improve the manuscript:

 1. Redesign the experiments as I suggested.

 2. More observations (at least 4) for each experiment are needed.

 3. Proper statistical analysis is essential. Simple t test is not enough as the experiment are in combination of different agents.

I have read this submission. I believe that I have an appropriate level of expertise to confirm that it is of an acceptable scientific standard, however I have significant reservations, as outlined above.

## References

[1] KunduASinghG: Dopamine synergizes with caffeine to increase the heart rate of Daphnia. *F1000Research*.2018;7: 10.12688/f1000research.12180.1 10.12688/f1000research.12180.1 PMC602423430002818

# Peer review: Referee response for version 1

**DOI:** 10.5256/f1000research.13184.r31347

**Published:** 2018-03-09

**Authors:** Rafael Antonio Vargas

**Affiliations:** 1 Departamento de Ciencias Fisiológicas, Facultad de Medicina, Pontificia Universidad Javeriana, Bogotá, Colombia

The original article by Kundu and Singh focuses on the study the effects of cardiovascular drugs on the heart rate of Daphnia. The authors have used an interesting heart model: Daphnia. This model has some advantages in comparison with classical animal models such as rats, mice, dogs, cats, and others. This model could be interesting for researchers from undeveloped countries.

 The study shows the effect of dopamine, caffeine, and alcohol on Daphnia heart rate (HR), which has been studied in other animal models. In this case, it is shown that dopamine and caffeine increase HR, and alcohol reduces HR. Interestingly, dopamine restores the low HR ethanol-induced. The authors claim that it’s probably similar effects of this agents in humans, however, the Daphnia heart does not represent a direct analog of mammalian cardiac function, so the interpretation of the results of the present study in terms of mammals is problematic.

 I have two comments/questions:

1. It is mentioned in the introduction, that atropine and atenolol decrease HR and this is not true in humans: Atenolol, a beta blocker reduces HR, but atropine, a cholinolytic agent, increase HR, so this statement must be revised.
2. It could be useful if the authors add the how HR was performed.

In general, after reading this submission, I consider that this is a short and interesting article about a simple model which will be useful for students, young researchers interested in alternatives to study cardiovascular function. After clarifying the comments the paper can be indexed.

I have read this submission. I believe that I have an appropriate level of expertise to confirm that it is of an acceptable scientific standard.

## References

[1] DasG: Therapeutic review. Cardiac effects of atropine in man: an update. *Int J Clin Pharmacol Ther Toxicol*.1989;27(10) :473-7 2684867

[2] TakahashiHFukuyamaMYonedaSOkabayashiHYoshimuraM: Comparison of nisoldipine and atenolol in the treatment of essential hypertension. *Arzneimittelforschung*.1989;39(3) :379-82 2667523
